# Determination of the rat estrous cycle vased on EfficientNet

**DOI:** 10.3389/fvets.2024.1434991

**Published:** 2024-07-23

**Authors:** Xiaodi Pu, Longyi Liu, Yonglai Zhou, Zihan Xu

**Affiliations:** ^1^Reproductive Section, Huaihua City Maternal and Child Health Care Hospital, Huaihua, China; ^2^Shenyang Institute of Computing Technology, Chinese Academy of Sciences, Shenyang, China; ^3^University of Chinese Academy of Sciences, Beijing, China; ^4^College of Biological Sciences, China Agricultural University, Beijing, China

**Keywords:** deep learning, EfficientNet, histopathology, estrus cycle, estrus staging, pathological image, classification

## Abstract

In the field of biomedical research, rats are widely used as experimental animals due to their short gestation period and strong reproductive ability. Accurate monitoring of the estrous cycle is crucial for the success of experiments. Traditional methods are time-consuming and rely on the subjective judgment of professionals, which limits the efficiency and accuracy of experiments. This study proposes an EfficientNet model to automate the recognition of the estrous cycle of female rats using deep learning techniques. The model optimizes performance through systematic scaling of the network depth, width, and image resolution. A large dataset of physiological data from female rats was used for training and validation. The improved EfficientNet model effectively recognized different stages of the estrous cycle. The model demonstrated high-precision feature capture and significantly improved recognition accuracy compared to conventional methods. The proposed technique enhances experimental efficiency and reduces human error in recognizing the estrous cycle. This study highlights the potential of deep learning to optimize data processing and achieve high-precision recognition in biomedical research. Future work should focus on further validation with larger datasets and integration into experimental workflows.

## 1 Introduction

Understanding the physiological and behavioral changes that occur in female animals at different stages of the estrous cycle is critical for interpreting experimental data. Female animals, including rats, experience significant variations in biological aspects such as gene expression (1), protein levels (2, 3), electrophysiological properties (4, 5), behavior (6, 7), and drug responses (8) due to their estrous cycle. Recognizing this, the National Institutes of Health (NIH) emphasized in 2015 the necessity to consider sex as a variable in studies and to include both male and female animals (9).

Rats are widely used in research due to their ease of care and short reproductive cycle. The estrous cycle, also known as the cycle, is the period of time between the onset of two estrus (10). As model organisms, rats play a key role in biomedical research. By studying rats, scientists have been able to gain insight into the mammalian reproductive system, revealing fundamental mechanisms related to the cell cycle, hormonal regulation, and reproductive behavior. These studies have provided important insights into deepening our understanding of human reproductive health. Their estrous cycle, comprising four main phases—proestrus (P), estrus (E), metestrus (M), and diestrus (D) (11, 12), is particularly important for studies on the female reproductive system. Accurate determination of these phases is crucial, as each phase can exhibit different responses to medications and in disease modeling, enhancing the reliability and applicability of experimental data.

**Figure 1:**
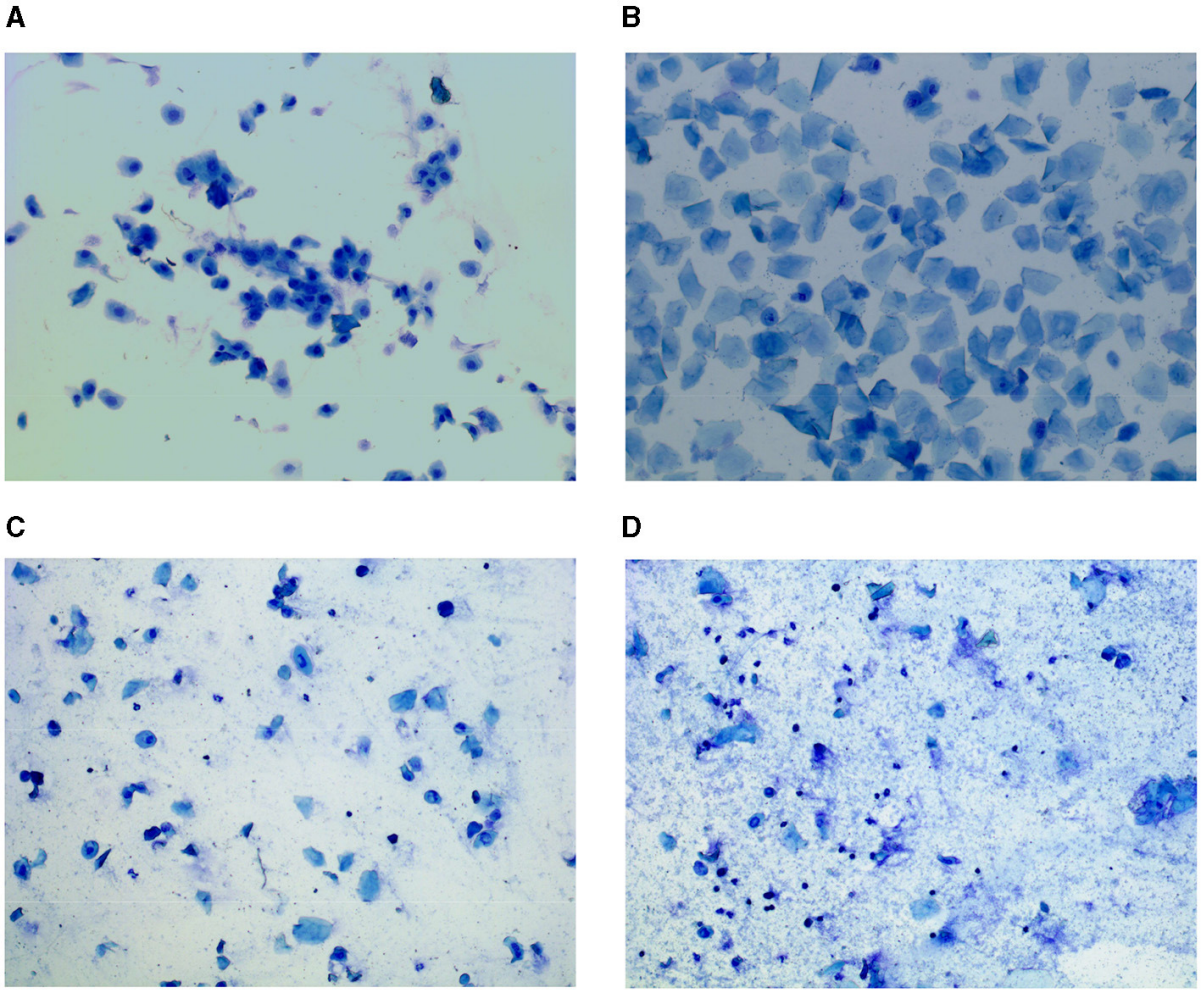
Vaginal cytology presenting each stage of the rat estrous cycle. Stages of the estrous cycle include the **(A)** proestrus, **(B)** estrus, **(C)** metestrus, and **(D)** diestrus.

In experiments, the stage of the estrous cycle in rats is usually determined by cytological analysis of vaginal smear samples. Specifically for each phase, the analysis is mainly based on the type, number, shape, and size of the cells in the smear, as well as their relative proportions to each other (13–17), as shown in Figures 1A–D. Briefly, the P Stage is in the follicular stage of development, with rising estrogen levels, lasting 12–14 h, and vaginal smears showing mainly nucleated cells and erythrocytes. The E Stage, in which rats show sexual receptivity, is the ovulatory stage and lasts for 9–15 h, with vaginal smears showing mainly keratinized cells. Immediately following the M Stage, which lasts 21–24 h, the corpus luteum begins to form in the ovary at this stage, estrogen levels decrease, and progesterone levels increase. Keratinized cells, nucleated cells, and leukocytes can be seen in the vaginal smear. D Stage has the longest cycle, lasting 55–57 h, in which the corpus luteum matures and secretes large amounts of progesterone to maintain the endometrium, and the vaginal smear shows mainly leukocytes (18, 19). Although vaginal cytological analysis is the traditional method for localizing the estrous cycle in rats, manual assessment has some limitations. For example, the process often requires prolonged training so that examiners can become proficient in recognizing the different phases; it is time-consuming and costly to manually identify estrous phases from images; and even professionally trained personnel may sometimes differ in their assessments. These limitations highlight the potential inconvenience and inconsistency of this approach.

Deep learning, especially research methods relying on artificial neural networks, has been widely used in several fields (20, 21), and the field of veterinary science and medicine is no exception (12, 22, 23). For this research area, we have conducted a comprehensive review of the relevant literature, with a special focus on the niche area of pathology. Pathology is the study of disease-induced changes in tissues and their causes. A large number of studies in the research literature based on convolutional neural network (CNN) technology have explored the composition and properties of various types of tissues through deep learning. For example, Sun et al. (24) classified endometrial tissue images and their findings classified pathological diagnoses into four categories (i.e., normal endometrium, endometrial polyp, endometrial hyperplasia, and endometrial adenocarcinoma) and achieved significant progress in classification results. In contrast, a hybrid convolutional deep neural network model developed by Yan et al. (25) focused on classifying breast cancer tumors using a wider range of image resources, demonstrating the potential of the model to be applied in this field.

In this research project, we applied deep learning algorithms to analyze stained samples collected from vaginal exfoliated cells. In this paper, we use the EfficientNet deep learning model for sample training and accurate classification of images. Currently, pathologists are still using microscopes to examine these digitally converted pathology samples. EfficientNet, as a cutting-edge image-processing network, is particularly well-suited to accurately identify and differentiate between various cell types from complex cellular images using its deep learning architecture (26). Adopting this approach effectively solves the problem of consistency associated with tedious training, time-consuming and subjective judgments required when manually determining the phases of the estrous cycle.The addition of EfficientNet enabled us to rapidly and accurately analyze large amounts of image data to accelerate the determination of the various phases of the estrous cycle, significantly improving the efficiency of the study and the reliability of the results (27, 28). During implementation, it was found that training using our proposed model took only 8–10 min, and the detection speed reached the second level, realizing significant time savings compared to traditional microscopy methods.

The main contributions are summarized as follows.

- In this study, we constructed a raw dataset dedicated to the study of vaginal decidual cell staining images for understanding the rat's estrous cycle, a collaborative research project spanning medicine, pathology, and computer science. By conducting research in this cutting-edge, cross-disciplinary field, we are able to explore in-depth aspects of microscopic image recognition and animal physiology.
- We used the EfficientNet model, which is usually used for target detection, as a classifier and achieved high accuracy in the identification of vaginal exfoliated cells and judgment of motility cycle, bridging the gap of research in this field. At the same time, such a model can provide a second opinion to pathologists to support their clinical diagnosis.
- We have made significant advances in fine-grained recognition by using the EfficientNet model to be able to extract more deep information from micrographs of vaginal exfoliated cells, further reducing the possibility of misjudging the motility cycle.

## 2 Materials and methods

### 2.1 Animals

At 8 weeks old, 40 female specific-pathogen-free (SPF) Sprague-Dawley (SD) rats were obtained from Changsha Tianqin Biotechnology Co., Ltd. in China. Following a 2-week acclimation period, 36 SD rats with stable estrous cycles were chosen for the study.

### 2.2 Data acquisition

In this paper, we used vaginal exfoliated cell staining to obtain microscopic images of the rat's estrous cycle. The specific operation was as follows: firstly, the cotton ball of the smaller end of the medical swab was completely moistened with saline, and the vaginal opening was fully exposed by gently lifting the rat's tail, and the moistened swab was gently put into the rat's vagina and slowly rotated for 2 or 3 times and then taken out; secondly, the swab taken out was evenly rotated and coated on an ordinary slide, with an area of about the size of the thumb's nail cap, and then the vaginal secretion on the slide was dried up and then stained with 0.2% methylene blue staining solution for about 15 min, and then gently rinsed with tap water. Then, after the slides were air-dried, an appropriate amount of neutral gum could be added to the slides, and then the slides were covered with a coverslip, avoiding the production of bubbles as much as possible, and then stored at room temperature; Lastly, each vaginal exfoliated cell smear was observed under a microscope and labeled. In order to obtain sufficient data, the experimental rats were injected with cyclophosphamide and leucovorin. Cyclophosphamide was used to induce immunosuppression, which can affect the estrous cycle and provide a model for studying the effects of immunosuppression on reproductive health. Leucovorin was administered to mitigate the toxic effects of cyclophosphamide. Data were collected every day for 4 consecutive weeks. In view of the fact that the rat's estrous cycle consists of four consecutive phases, namely, the pre-motility phase, the motility phase, the post-motility phase, and the inter-motility phase, the motility cycle was determined according to the presence and the number of keratinized epithelial cells, leucocytes, and nucleated epithelial cells on the vaginal smears, and the pictures were combined with the expert prior knowledge to correspond one-to-one with the motility cycle. The target classification results are labeled one-to-one, constituting the dataset for the EfficientNet model. Obtained 646 for P stage, 672 for E stage, 670 for M stage, and 667 for D stage, for a total of 2,267. The datasets used and/or analyzed during the current study available from the corresponding author on reasonable request.

### 2.3 Image preprocessing

Before training for deep learning, it is crucial to properly preprocess the collected image data related to the rat estrous cycle. This step ensures that our dataset is not only clean but also consistent, enabling the trained model to achieve optimal performance. Therefore, we adopted a series of standard operations for data preprocessing: first, in the image quality control stage, all microscopic images were carefully examined to exclude those samples whose image quality was degraded due to improper sampling, focusing errors, or motion blur. The remaining images were subjected to an initial judgment of cellular structure recognition, with the aim of obtaining training samples for the model, and samples that contributed substantially to the subsequent analysis were retained.

Subsequently, image enhancement techniques were applied to enhance the details of the images. By adjusting the brightness and contrast, we enhanced the visual contrast of the cellular structures to provide clear inputs for subsequent modeling. Similarly, the sharpening process further enhances the recognition of local details without losing the realism of the image. Image format conversion is another part of the preprocessing, and in this step we strictly control the conversion quality loss by uniformly converting all images to JPEG format suitable for processing by the machine learning framework. This processing also includes a uniform resizing of the images, which ensures that each image meets the input specifications of the training model (29, 30), as shown in the following equation, where the image data is uniformly resized to 224 dimensions. The original image also maintained to preserve key biological feature information.

In order to scale the image from the original resolution to the new resolution, we use Equation (1) to (4). Firstly, we need to calculate the scale factor


(1) $$\begin{array}{l} \begin{array}{c} s_{w}=\frac{W_{\text{new}}}{W_{\text{orig}}}\\ s_{h}=\frac{H_{\text{new}}}{H_{\text{orig}}} \end{array} \end{array}$$

We want to keep the aspect ratio of the image constant, choose the smaller of the width and height scale factors as the uniform scale factor:


(2) $$\begin{array}{l} s=\min\left(s_{w},s_{h}\right) \end{array}$$

This scale factor is then used to calculate the scaled width and height:


(3) $$\begin{array}{l} \begin{array}{c} W_{\text{scaled}}=s\times W_{\text{orig}}\\ H_{\text{scaled}}=s\times H_{\text{orig}} \end{array} \end{array}$$

If the scaled width and height do not reach the target resolution, the target resolution can be reached by padding (padding). We use zero padding, the amount of padding can be calculated as follows:


(4) $$\begin{array}{l} \begin{array}{c} \text{pad\_width}=\frac{W_{\text{new}}-W_{\text{scaled}}}{2}\\ \text{pad\_height}=\frac{H_{\text{new}}-H_{\text{scaled}}}{2} \end{array} \end{array}$$

Because we need to fill an odd number, we can adjust the fill amount slightly so that the left and right (top and bottom) fills are as even as possible.

In order to further enhance the generalization ability of the model and reduce the possibility of overfitting, data enhancement means were applied to the image dataset, including rotation, scaling, flipping, and cropping of the images. These operations generated variants of the training images, thus simulating experimental environments with high diversity. Finally, to ensure the accuracy and validity of the model evaluation, we divided the entire dataset into training, validation, and testing sets, with uniformly distributed samples in each subset to ensure fairness and comprehensiveness of the evaluation.

### 2.4 EfficientNet model

**Figure 2:**
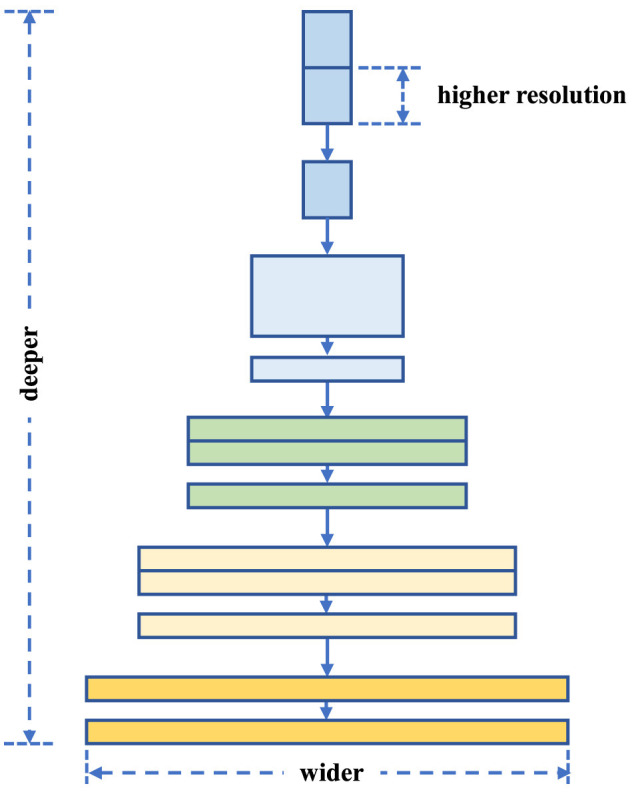
A composite scaling method for feature extraction strategy.

We use the EfficientNet model as a classifier based on the convolutional neural networkCNN (CNN) architecture. EfficientNet is an advanced CNN architecture that was first proposed by Tan and Le in their 2019 paper (31). The core concept of EfficientNet lies in equalizing the depth, width, and image resolution, which are systematically coordinated through a method known as the compound scaling method (32). Traditional CNN architectures are designed with a given resource budget and scaled to improve accuracy as more resources become available (33). However, this simple scaling is often done for a single dimension, either by increasing the width of the network (the number of neurons in a layer), or the depth of the network (the number of layers), or by increasing the resolution of the input image.EfficientNet proposes a different approach: the composite scaling method is able to take into account all three dimensions at once, as shown in Figure 2, and to unify scaling by a fixed scale factor ϕ to unify the scaling, as shown in the following Equation (5), as a way to maintain the balance between the dimensions. This balanced approach proves to be effective in improving the performance of the model while maintaining high efficiency of the parameters.


(5) $$\begin{array}{l} \text{ depth: }d=\alpha^{\phi }\\ \text{ width: }w=\beta^{\phi }\\ \text{ resolution: }r=\gamma^{\phi }\\ \text{ s.t. }\alpha \cdot \beta^{2}\cdot \gamma^{2}\approx 2\\ \alpha \geq 1,\beta \geq 1,\gamma \geq 1 \end{array}$$

Where, *w*, *d*, *r* are the scaling coefficients of the scaling network for width, depth, and resolution, respectively; α, β, γ are all constants obtained from a very small range of network searches; intuitively, ϕ is a specific coefficient that controls the amount of resources used for a resource, and α, β, γ determines exactly how the resource is allocated.

The EfficientNet model adopts the mobile bottleneck convolution (MBConv) of MobileNet V2 (34) as its core network architecture and draws on the squeeze-and-excitation technique of SENet (35) to further optimize the network architecture. The details of the EfficientNet architecture adopted in this study are demonstrated in Table 1. The starting phase, i.e., Phase 1, uses a standard convolutional layer of 3x3 size with a step size of 2; from Phase 2 to Phase 8, a duplicate MBConv structure is used for stacking; and Phase 9, consists of a standard convolutional layer of 1x1 size, an average pooling layer, and a fully-connected layer.

**Table 1:** Description of each stage in the network architecture.

**Stage**	**Operator**	**Resolution**	**Channels**	**Layers**
1	*Conv*3 × 3	224 × 224	64	1
2	*MBConv*1, *k*3 × 3	112 × 112	32	1
3	*MBConv*6, *k*3 × 3	112 × 112	48	2
4	*MBConv*6, *k*5 × 5	56 × 56	80	2
5	*MBConv*6, *k*3 × 3	28 × 28	160	3
6	*MBConv*6, *k*5 × 5	14 × 14	224	3
7	*MBConv*6, *k*5 × 5	14 × 14	384	4
8	*MBConv*6, *k*3 × 3	7 × 7	640	4
9	*Conv*1 × 1& Pooling & FC	7 × 7	2,560	1

## 3 Experimental results

### 3.1 Performance indicators

In this study, a set of specific evaluation criteria was established to determine the performance of our model for detection during the estrous cycle phase. Therefore, we chose metrics such as accuracy, precision, recall, and F1 score for effectiveness assessment. Among them, the confusion matrix (12) serves as a tool, which can be constructed based on the values of the parameters mentioned above, to evaluate the success of the classification algorithm.

Accuracy is a key measure of how correct the algorithm is in performing the classification (36). The resulting accuracy value is derived by comparing the ratio of the number of correctly classified samples to the total number of samples. It is often used as an important measure of the model's performance. The value of the accuracy rate is between 0 and 1. The closer the value is to 1, the higher the classification success rate of the model (37). The specific Equation (6) is demonstrated below.


(6) $$\begin{array}{l} \text{Accuracy}=\frac{TP+TN}{TP+FN+FP+TN} \end{array}$$

where TP denotes True Positives, i.e., true class positive instances; TN denotes True Negatives, i.e., true class negative instances; FP denotes False Positives, i.e., false positive instances; and FN denotes False Negatives, i.e., false negative instances.

The assessment metric of Precision is primarily used to measure the ability of a neural network to correctly identify positive samples (38). It is specifically defined as the ratio of the number of true positive predictions of the model to the number of all positive predictions. As this ratio rises, the probability of successful recognition by the network increases. The corresponding calculation is shown in the following Equation (7).


(7) $$\begin{array}{l} \text{Precision}=\frac{TP}{TP+FP} \end{array}$$

where TP denotes True Positives, i.e., true class positive instances; FP denotes False Positives, i.e., false positive instances.

The metric Recall measures the proportion of samples correctly identified by the model as belonging to their respective classes. Specifically, it quantifies how many of the actual positive samples are correctly predicted as positive, and how many of the actual negative samples are correctly predicted as negative. This metric is crucial for assessing how well the model identifies samples in both positive and negative categories. The Equation (8) for recall is shown below.


(8) $$\begin{array}{l} \text{Recall}=\frac{TP}{TP+FN} \end{array}$$

where TP denotes True Positives, i.e., true class positive instances; FN denotes False Negatives, i.e., false negative instances.

The F1 Score is a metric that combines precision and recall, providing a single measure that reflects the balance between these two metrics. It is particularly useful in scenarios where we want to consider both the completeness (recall) and correctness (precision) of the model's predictions. The Equation (9) for F1 Score integrates these components to give a comprehensive assessment of the model's performance.


(9) $$\begin{array}{l} \text{F}1\text{ Score}=\frac{2*\left(\text{Precision}*\text{Recall}\right)}{\left(\text{Precision}+\text{Recall}\right)} \end{array}$$

### 3.2 Model setting

In deep learning, model training is both complex and computationally intensive. By deploying Graphics Processing Unit (GPU) acceleration in our daily research or development environments, we can significantly improve the efficiency of model training and quickly achieve better training results. For all experiments, NVIDIA Corporation (NVIDIA) 3050ti GPU was used for model training and testing. In addition, in order to optimize the model training, Adam was chosen as the optimization algorithm in this study, and the training Epochs were set to 100, the initial learning rate was set to 0.001, and the batch size was adjusted to 16, and the detailed configurations can be found in Table 2. In addition, it is worth mentioning that the use of GPU acceleration is especially critical for dealing with large-scale datasets and complex model architectures, which not only reduces the training time, but also helps the experiments to iterate rapidly and optimize the model performance.

**Table 2:** Training parameter values for EfficientNet model.

**Parameters**	**Value**
Rate of learning	0.001
Number of epochs	100
Batch size	16
Image size	224
Optimization algorithm	Adam

### 3.3 Discussion and results

**Figure 3:**
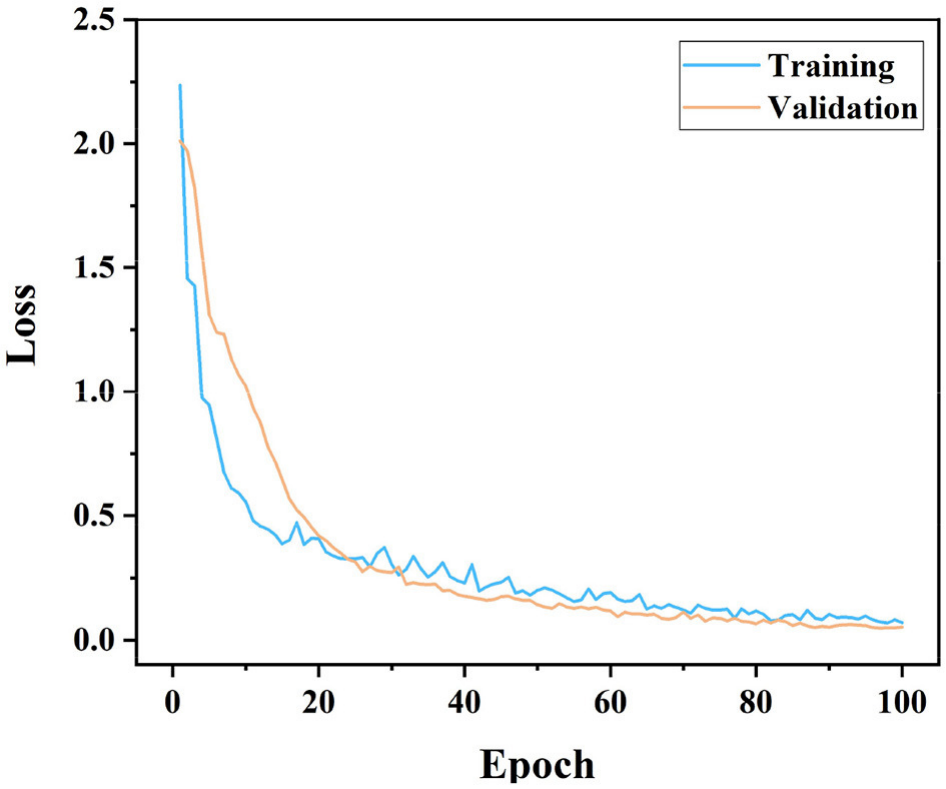
Training-validation loss graphs according to the EfficientNet model.

As can be seen in Figure 3, the model loss (a metric characterizing poor model performance) drops rapidly from a high value of slightly above 2.5 in the early stages of training, indicating that the EfficientNet model quickly begins to learn and improve its performance in the application of rat motility classification. The training and validation loss curves are very close to each other and remain consistent over time, indicating that the model has good generalization ability, i.e., it can be effectively applied to unseen data, and also that there is no overfitting. After about 50 epochs, the two loss curves are essentially stable with only minor fluctuations, which means that the model may be approaching the limit of its learning potential. At this stage, the performance improvement of the model becomes marginal, and further training may not result in significant performance gains unless the model configuration or training process is changed. The loss value eventually stabilizes at around 0.4, a point that indicates that the EfficientNet model performs well on the task of classifying rat motility on vaginal micro-smears.

**Figure 4:**
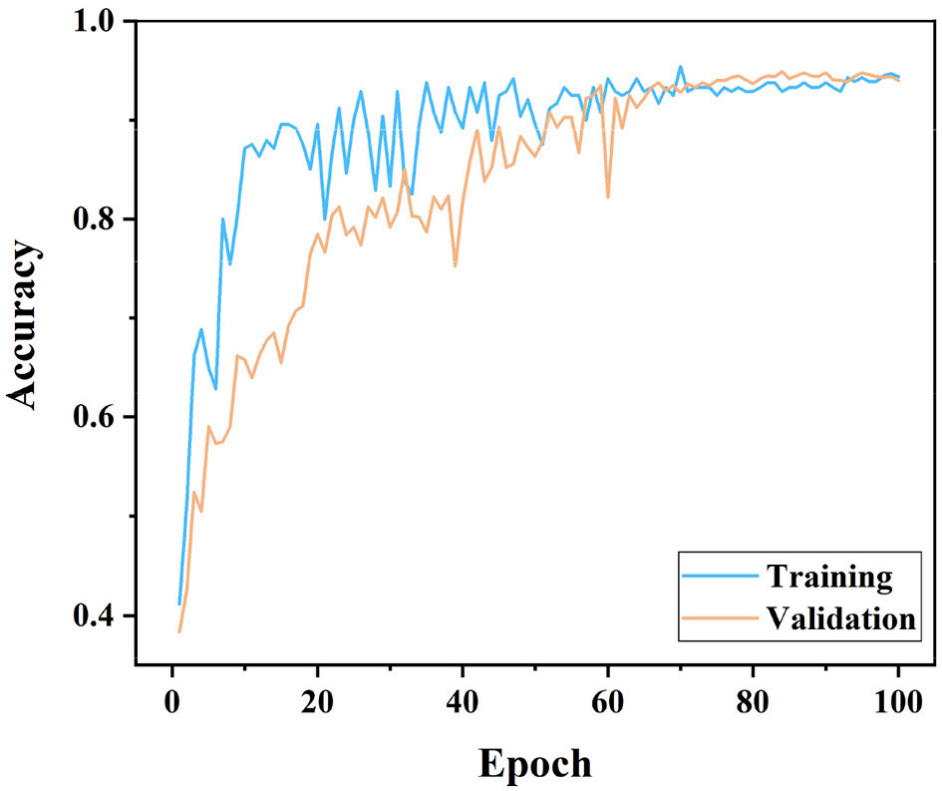
Training-validation accuracy graphs according to the EfficientNet model.

As can be seen in Figure 4, both training and validation accuracies seem to increase from about 0.4 when starting from an Epoch of 0. This indicates that initially the model learns from a base level and improves its performance very quickly, which is consistent with the early behavior of typical deep learning model training curves. It is worth noting that the training curve increases faster than the validation curve and reaches an accuracy close to 1.0 earlier. As can be seen from the curves, the training curve has more fluctuations than the validation curve. This can be due to the fact that the model encounters some difficult to fit data during the learning process. The validation curve is usually smoother, but overall the accuracy is lower than the training set. It does not reach the high point of the training accuracy and shows some degree of generalization.

Based on the analysis results of these two figures, it is evident that the EfficientNet model has good learning ability and generalization performance in dealing with specific biomedical image classification problems, showing its advantages in complex image recognition tasks.

Table 3 shows the distribution and evaluation metrics of the results obtained from the data trained on the EfficientNet model. It can be seen that the overall accuracy of the EfficientNet model is 95.8% and the F1 score, which is the reconciled mean of precision and recall, is 0.968, indicating a good balance between precision and recall. In terms of category stage performance: the Proestrus stage, the model performs slightly lower than the overall in this stage, with an accuracy of 97.0%, a precision of 0.933, a recall of 0.949, and an F1 score of 0.941. The precision is lower than in the other stages, indicating that there are more false positives; the Estrus stage: the model performs very well in this stage, having the highest accuracy (99.1%) and perfect recall (1.0). It correctly identifies all the actual positive examples with the highest F1 score of all phases at 0.983; Metestrus phase: here the model has 98.2% accuracy, very high precision (0.983) and recall (0.950). The F1 score of 0.966 is still very high and shows a balanced performance; Diestrus phase: performance is Estrus stage, with 99.1% accuracy, 0.983 precision, 0.983 recall, and an F1 score of 0.983. the model has a very high ability to correctly recognize and classify pictures in this stage.

**Table 3:** Test results of data trained with EfficientNet model.

**Stage**	**Number of images**	**Accuracy (%)**	**Precision**	**Recall**	**F1 score**
Whole	239	95.8	0.967	0.971	0.968
Proestrus	59	97.0	0.933	0.949	0.941
Estrus	58	99.1	0.967	1.0	0.983
Metestrus	62	98.2	0.983	0.950	0.966
Diestrus	60	99.1	0.983	0.983	0.983

**Figure 5:**
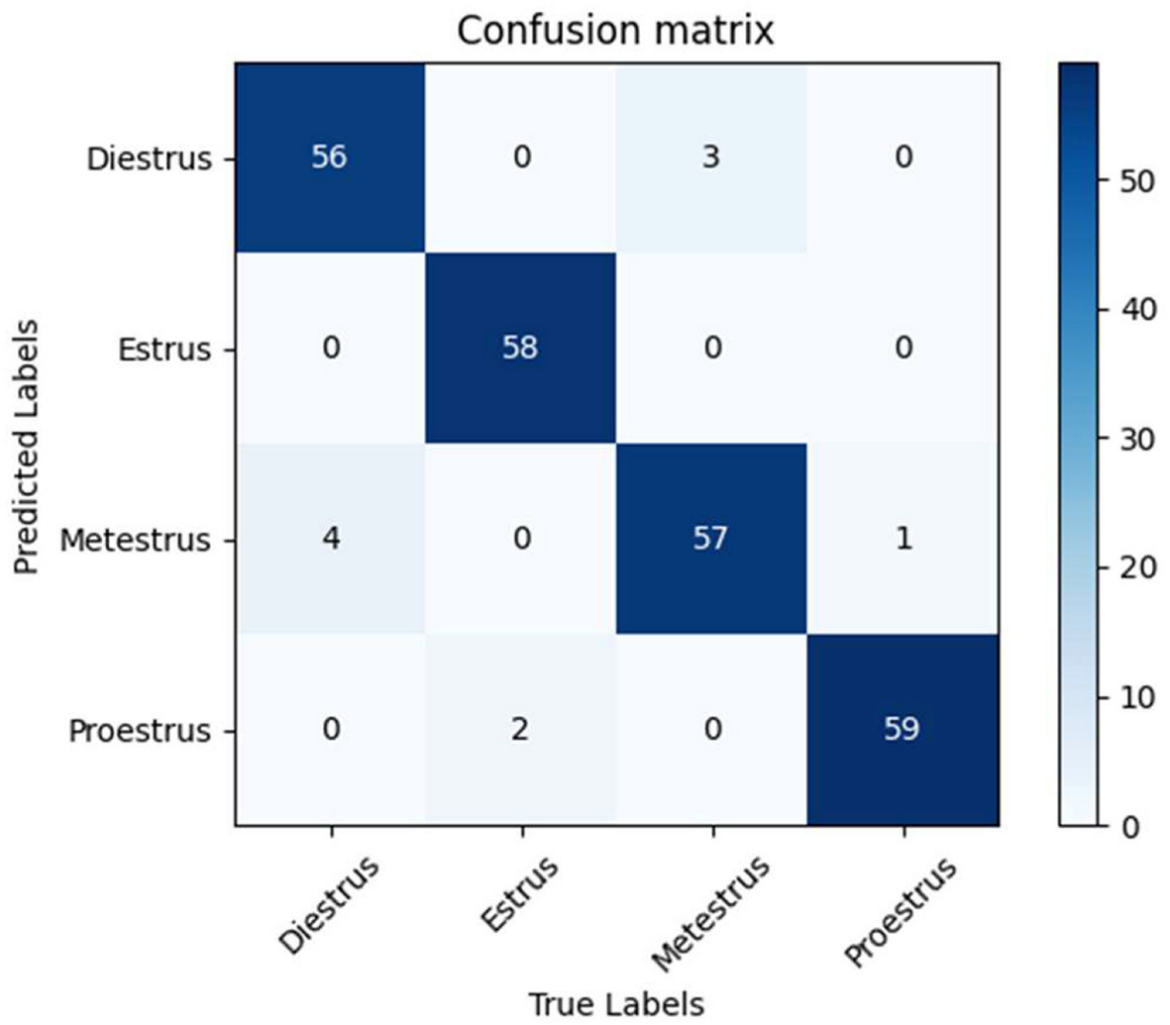
Confusion matrices for the EfficientNet model.

Based on the results of the confusion matrix in Figure 5, we can see that the performance of this classification model in recognizing the four categories; Diestrus, Estrus, Metestrus and Proestrus is good. Specifically, for the Diestrus category, the model correctly identified 56 cases, but three cases were misidentified as Metestrus; in the Estrus category, the model performs well, correctly identifying all 58 cases without misidentification; for the Metestrus category, the model correctly identifies 57 cases, but four cases were misidentified as Diestrus and one case was misidentified as Proestrus; in the Proestrus category, the model also performed well, correctly identifying 59 cases, but two cases were misidentified as Estrus. in addition, the model predicted Estrus and Proestrus very accurately, with no misidentifications to other stages. There was some confusion between Diestrus and Metestrus, especially Metestrus was misidentified as Diestrus more often, which may be due to the greater similarity in features between these two categories. At the same time, there was also some confusion between Metestrus and Proestrus, albeit in small numbers, which indicates that the model may need further improvement in distinguishing between these two stages.

Overall, the model's performance on the classification task remains reliable, with most predictions being correct. However, in order to further improve the model's performance, focusing on reducing misclassification between Diestrus and Metestrus, as well as the few confusions between Estrus and Proestrus, and Metestrus and Proestrus, may be the focus of the next improvement efforts.

## 4 Conclusion

In this study, the EfficientNet deep learning model was successfully introduced to the field of biomedical image analysis. In particular, the model was used for the first time to identify and classify vaginal microscopic smears of rats during the estrous cycle on a large scale and in an automated manner. In the four key stages of Proestrus, Estrus, Metestrus, and Diestrus, EfficientNet showed excellent recognition ability, achieving a high accuracy of 95.8%, as well as F1 scores of more than 0.94 in all stages, especially in the Estrus and Diestrus stages. This breakthrough innovation not only demonstrates the great potential of EfficientNet in the field of biomedical image processing, but also provides a reliable methodological tool for subsequent related research.

## Data availability statement

Missing values, inconsistent data, and erroneous records may exist in the data set. These issues may affect the accuracy of analysis results. Requests to access these datasets should be directed to: xiaodipu2022@163.com.

## Ethics statement

The animal study was approved by Ethics Committee of Huaihua Maternal and Child Health Hospital. The study was conducted in accordance with the local legislation and institutional requirements.

## Author contributions

XP: Writing – original draft. LL: Validation, Investigation, Writing – review & editing. YZ: Conceptualization, Writing – review & editing. ZX: Supervision, Formal analysis, Visualization, Writing – review & editing.
